# Association between genetic polymorphisms and carotid atherosclerosis in patients treated with radiotherapy for nasopharyngeal carcinoma

**DOI:** 10.1186/s13014-015-0341-8

**Published:** 2015-02-13

**Authors:** Chuang Yuan, Shea Ping Yip, Vincent WC Wu, Dora LW Kwong, Isabella WY Cheuk, Michael Ying

**Affiliations:** Department of Health Technology and Informatics, The Hong Kong Polytechnic University, Hung Hom, Kowloon, Hong Kong, SAR China; Department of Clinical Oncology, The University of Hong Kong, Pokfulam, Hong Kong, SAR China; Current address: Medical Research Center, Changsha Central Hospital, Changsha, Hunan China

**Keywords:** Radiation, Nasopharyngeal carcinoma, Carotid atherosclerosis, Carotid plaque score, Single nucleotide polymorphisms, Paraoxonase

## Abstract

**Background:**

Radiotherapy (RT) of the neck is commonly given to nasopharyngeal carcinoma (NPC) patients for preventing cervical lymph node metastasis. However, neck RT may induce the development of carotid atherosclerosis. The mechanisms of radiation-induced carotid atherosclerosis are still unclear and no previous study has investigated the genetic involvement of radiation-induced carotid atherosclerosis. The present study aims to determine the association between genetic polymorphisms and carotid atherosclerosis in patients treated with RT for nasopharyngeal carcinoma.

**Methods:**

The present study recruited 128 post-RT NPC patients. Carotid plaque score was assessed using ultrasonography. Thirteen single nucleotide polymorphisms (SNPs) that affect the function of anti-atherosclerotic genes, including *SOD2, SOD3, CAT, PON1, PPARG, ADIPOQ, IL10, TGFB1* and *NOS3*, were genotyped. Association between the 13 SNPs and carotid atherosclerosis was evaluated using multiple regression after adjustment for covariates (PLINK). Multiple testing was corrected using Benjamini-Hochberg step-up false discovery rate controlling procedure.

**Results:**

rs662 and rs705379 of *PON1* were close to be significantly associated with carotid plaque score (Corrected *P* value, *P*_*cor*_ = 0.0528 and *P*_*cor*_ = 0.0842). When the two SNPs were combined together, TC haplotype in rs662-rs705379 of *PON1* was significantly associated with higher carotid plaque score (*P*_*cor*_ < 0.05). None of the other SNPs showed significant association with carotid plaque score.

**Conclusions:**

TC haplotype in rs662-rs705379 of *PON1* is likely to be a genetic risk factor of carotid plaque score. Post-RT NPC patients with the TC haplotype may need earlier and more frequent carotid ultrasound examinations for early detection of carotid atherosclerosis.

## Background

Nasopharyngeal carcinoma (NPC) is a common head and neck malignancy in Southeast Asia and Southern China [1]. Radiotherapy (RT) is the standard strategy for treating nasopharyngeal carcinoma (NPC). Owing to the high prevalence of cervical lymph node metastasis in NPC patients, RT of the neck is usually given to the patients for preventing or treating the nodal metastasis [2]. However, ionizing radiation in neck RT damages the carotid artery and may induce carotid atherosclerosis, which may lead to cerebrovascular events [3-6].

The mechanisms of radiation-induced carotid atherosclerosis are still unknown. However, the mechanisms of spontaneous atherosclerosis are well established, which provide baseline information for the understanding of the mechanisms of radiation-induced carotid atherosclerosis. Different pathways in the regulation of oxidative stress, lipid metabolisms, and inflammation may protect the carotid artery from atherosclerosis. There are many genes whose encoded proteins are involved in these protective pathways. Superoxide dismutases (SODs) are the primary enzymes in the defense of oxidative stress, which convert the toxic superoxide anions to the less toxic hydrogen peroxide (H_2_O_2_) [7]. Catalase (CAT) further scavenges the toxic H_2_O_2_ by converting it into water (H_2_O) and molecular oxygen (O_2_) [8]. Paraoxonase 1 (PON1) prevents the oxidation of low intensity lipoprotein (LDL), and inhibits the uptake of oxidized LDL by and cholesterol synthesis in macrophages [9]. Peroxisome proliferators-activated receptor γ (PPARG) is a pivotal nuclear receptor that regulates the expression of genes involved in lipid metabolisms and inflammatory responses [10]. Adiponectin (ADIPOQ) suppresses the inflammatory responses and the uptake of oxLDL by macrophages [11]. Interleukin-10 (IL10) and transforming growth factor-β1 (TGFB1) are the most important anti-inflammatory cytokines in immune cells [12,13]. Endothelial nitric oxide synthase (NOS3) catalyzes the production of nitric oxide, which prevents platelet aggregation, adhesion molecule expression in endothelial cells and vascular SMC proliferation [14].

Some single nucleotide polymorphisms (SNPs) that affect the expression of these genes (*SOD2, SOD3, CAT, PON1, PPARG, ADIPOQ, IL10, TGFB1* and *NOS3*) and/or the functions of their corresponding proteins have been shown to be associated with spontaneous atherosclerosis [15-24]. However, the association of these SNPs with radiation-induced carotid atherosclerosis is still unknown. Therefore, the present study was undertaken to investigate the association between the SNPs in these nine genes and the severity of carotid atherosclerosis in post-RT NPC patients. The findings will offer potential genetic markers of radiation-induced carotid atherosclerosis, which might facilitate the selection of high-risk patients with carotid atherosclerosis so that timely diagnosis and treatment can be given to the patients.

## Methods

### Subjects

Post-RT NPC patients were recruited from the Department of Clinical Oncology of Queen Mary Hospital from March 2013 to March 2014. The inclusion criteria of subjects were local residents, Han Chinese NPC patients, older than 18 years, and completed RT for at least four years, whilst the exclusion criteria of subjects were more than one course of RT, history of carotid atherosclerosis prior to RT, previous carotid endarterectomy and carotid stenting.

This study was approved by the Human Subject Ethics Subcommittee of the Hong Kong Polytechnic University and the Institutional Review Board of the University of Hong Kong/Hospital Authority Hong Kong West Cluster. Written consent was obtained from all patients before the commencement of the interview and ultrasound examination.

### Clinical information

Archived clinical records were reviewed and individual face-to-face interviews were conducted. The information of post-RT duration, radiation dose, chemotherapy and history of carotid atherosclerosis was obtained from archived clinical records. The presence of cardiovascular risk factors was identified as follows: 1) DM, diagnosed with DM in the clinical record, taking medications to lower blood glucose and/or fasting plasma (blood) glucose ≥ 7.0 (6.1) mmol/L [25]; 2) hypertension, diagnosed with hypertension in the clinical record, undergoing anti-hypertensive medications and/or the measured blood pressure ≥ 140/90 mmHg [26]; 3) hypercholesterolemia, diagnosed with hypercholesterolemia in the clinical record, undergoing medications to lower the cholesterol level and/or fasting total cholesterol ≥ 5.2 mmol/L [27]; 4) CHD, diagnosed with coronary vascular disease in the clinical record and/or had coronary stenting [28]; 5) smoking, current smoker consuming 10 cigarettes per day for at least six months [28].

### Selecting and genotyping of SNPs

A literature research was performed for the selection of candidate genes and relevant SNPs in the present study (Pubmed). Candidate genes involving oxidative stress, lipid metabolism and inflammation and having association with atherosclerosis were reviewed. Only the genes, in which SNPs influence the expression of these genes or the function of the encoded proteins, and have minor allele frequency > 0.1 in Han Chinese and evidences for association with spontaneous atherosclerosis, including carotid atherosclerosis, coronary atherosclerosis, and ischemic cardiovascular and cerebrovascular diseases, were selected. In total, 13 SNPs in the 9 genes, *SOD2*, *SOD3*, *CAT*, *PON1*, *PPARG*, *ADIPOQ*, *IL10*, *TGFB1* and *NOS3,* were included in the present study (Table 1) [15-24]. Genomic DNA was extracted from 6 ml of peripheral blood for genotyping. Restriction fragment length polymorphism (RFLP) and unlabeled probe melting analysis (UPMA) were used for genotyping as described previously [29-31]. The primers and probes used in genotyping are shown in Table 1.

**Table 1:** **Primers and probes in genotyping**

**Genes**	**SNPs**	**Forward primers**	**Reward primers**	**Probes**	**Genotyping methods**
*SOD2*	rs4880	CCA GCC TGC GTA GAC GGT	CGT GGT GCT TGC TGT GGT G	AGC CCA GAT ACC CCA AAG CCG GAG CCA Gaa	UPMA
*SOD3*	rs2536512	AGG ACT CAG CGG AGC CCA ACT	18 T + TTG GCA CGG GAC GCA AGC TG	-	RFLP (BstUI)
*CAT*	rs769217	TGT TAC CGC CCC TAG TCA GTG TC	CAC CTC GGG AGC ACC TTT ACC A	-	RFLP (BstXI)
*PON1*	rs662	10 T + GAA TGA TAT TGT TGC TGT GGG ACC TGA G	CCA TCG GGT GAA CTG TTG ATT CCA TTA G	-	RFLP (MboI)
	rs705379	AAA TGG GAC TTT TGG CTG A	ACA CTG ACG GGC TAG GA	CGC CGA TTT GCC CGC CCC GCC CCT CCC Caa ta	UPMA
*PPARG*	rs3856806	GCT GAA CCA TCC TGA GTC CTC	33 T + TGG AAG AAG GGA CAT GTT GGC	-	RFLP (NspI)
*ADIPOQ*	rs1501299	CAC CGA CAG AGC CTT GCA CAT TAG	GGG GGT CTG CAC AGG TTG GAT G	-	RFLP (BsmI)
	rs2241766	15 T + GAT GCT GTT GCT GGG AGC TGT	30 T + CCC GAG A CGC CAT CCA ACC TGT GC	-	RFLP (AvaI)
*IL10*	rs1800872	GGA GCC TGG AAC ACA TCC TG	AAA TGA GGG GGT GGG CTA AAT ATC	ACC CCC CCT GTC CTG TAG GAA GCC AGt CTa ata	UPMA
*TGFB1*	rs1800469	TGA CCC CAG CTA AGG CAT G	TTT TTC CTC TTC TCC CGA CCA G	-	RFLP (Eco81I)
	rs1800470	TCA CCA GCT CCA TGT CGA TA	ATC CCT GTT CGC GCT CTC	CAG CAG CGG TAG CAG CAG CGG CAG CAG Caa	UPMA
	rs4803455	TCA CTG CAA CCT CTG TGT CTT	20 T + CTG CAT ATT TGA CAC CCT GTA TT	-	RFLP (TasI)
*NOS3*	rs1799983	ACC CCA GAA AAC GGT CGC TTC G	CCC CGA TTT CCA GCA GCA TGT TG	-	RFLP (MboI)

UPMA, unlabeled probe melting analysis; RFLP, restriction fragment length polymorphism.

### Ultrasound examinations

**Figure 1:**
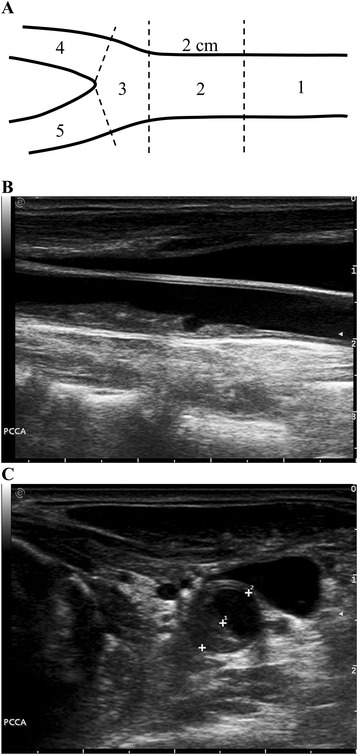
**Assessment of carotid plaque score. A)**. Five segments of the extra-cranial carotid artery: 1. proximal common carotid artery (≥2 cm proximal to carotid bifurcation); 2. distal common carotid artery (<2 cm proximal to carotid bifurcation); 3. carotid bulb and bifurcation; 4. internal carotid artery; and 5. external carotid artery. **B)**. A longitudinal gray-scale image of a carotid plaque in the distal common carotid artery. **C)**. A transverse gray-scale ultrasound image of the carotid plaque in B. The degree of the carotid stenosis is expressed as the percentage reduction of lumen diameter (1-D1/D2 = 41.3%). Carotid plaque score is the summation of the degree of carotid stenosis of the five segments in both carotid arteries.

Carotid ultrasound examinations were performed in a 22°C air-conditioned examination room using the Esaote MyLab Twice ultrasound unit in conjunction with a 4–13 MHz linear transducer (Esaote, Genoa, Italy). Subjects lied supine on the examination couch with the neck slightly extended and the head turned away from the side under examination. Using gray-scale ultrasound, the extra-cranial carotid artery was screened longitudinally and transversely. Carotid plaque was identified as a focal thickening >50% of the adjacent intima-media layer [32]. Once a carotid plaque was identified, transverse gray-scale images of the plaque were obtained and the degree of carotid stenosis was expressed as a percentage reduction of the lumen diameter at the most stenotic site. Carotid plaque score was evaluated using an adjusted plaque scoring system [28]. In the scoring system, the carotid artery was divided into five segments: 1. Proximal common carotid artery (≥2 cm proximal to carotid bifurcation); 2. Distal common carotid artery (<2 cm proximal to carotid bifurcation); 3. Carotid bulb and bifurcation; 4. Internal carotid artery; and 5. External carotid artery. The degree of carotid stenosis in each segment was measured and carotid plaque score was expressed as the summation of the degree of carotid stenosis of all segments in both carotid arteries (Figure 1).

### Statistical analysis

Data of carotid plaque score was transformed logarithmically because it was not normally distributed. Testing of genotypes for Hardy-Weinberg equilibrium (HWE) in all subjects was determined by exact test as executed in PLINK (version 1.07, [33]). The threshold for significant deviation from HWE was set as 0.01 [34]. Only markers fulfilling HWE were included in association analyses.

In the potential covariates, such as age, gender, radiation dose, chemotherapy, post-RT duration and cardiovascular risk factors, the significant predictors in regression models were adjusted in association analyses. Linear regression executed in PLINK was used for assessing the association between single SNP and carotid plaque score with adjustment for post-RT duration and number of cardiovascular risk factors (significant predictors in regression models). The regression analysis was performed under additive, dominant or recessive models. FDR correction was used for correcting multiple testing. *P*_*cor*_ < 0.05 was considered as significant for association analysis.

Three genes, *PON1*, *ADIPOQ* and *TGFB1*, had more than one SNP examined in the present study. The haplotypes in each of these three genes were determined for the association with carotid plaque score in post-RT NPC patients. Sliding window (2 or 3 SNPs per window) using linear regression in PLINK was utilized for the association analysis with adjustment for post-RT duration and number of cardiovascular risk factors. FDR correction was also used for correcting multiple testing. Linkage disequilibrium (LD) statistics D’ and r^2^ in paired SNPs were calculated using Pairwise LD in PLINK. *P*_*cor*_ < 0.05 was considered as significant for association analysis.

## Results

### Demographic information

A total of 128 post-RT NPC patients were included in the present study. All patients were treated conventional 2D RT of the neck. The mean age of the patients was 55.2 ± 8.8 years with a range of 33 to 86 years. There were 86 males and 42 females. The mean radiation dose was 66.82 ± 3.20 Gy with a range of 58.44 to 73.72 Gy. Of the 128 patients, 63 were also treated with chemotherapy. The mean post-RT duration was 12.8 ± 6.0 years with a range of 4 to 37 years. The most common cardiovascular risk factor was hypercholesterolemia (n = 39), followed by hypertension (n = 35) and then by DM (n = 14). Only 5 patients were current smoker, and 5 patients had CHD and 7 patients developed stroke or transient ischemia attack (Table 2).

**Table 2:** **Demographic information of post-RT NPC patients**

**Parameters**	**Total n = 128**
Age, years	55.2 ± 8.8
Gender (female/male), n	42/86
Chemotherapy, n (%)	63 (49.2%)
Radiation dose, Gy	66.82 ± 3.20
Post-RT duration, years	12.6 ± 6.0
Hypercholesterolemia, n (%)	39 (30.5%)
Hypertension, n (%)	35 (27.3%)
Diabetes mellitus, n (%)	14 (10.9%)
Current smoker, n (%)	5 (3.9%)
Coronary heart disease, n (%)	5 (3.9%)
Stroke or transient ischemia attack, n (%)	7 (5.5%)
Presence of carotid plaque, n (%)	114 (89.1%)
Carotid plaque score	1.41 ± 1.37

### Association analysis

Genotype proportions were all in HWE for 13 SNPs (*P* > 0.01, Table 3). In the 13 SNPs, only rs662 and rs705379 in *PON1* were close to be significantly associated with carotid plaque score in post-RT NPC patients (rs662, *P*_*cor*_ = 0.0842 in additive model and *P*_*cor*_ =0.0528 in dominant model; and rs705379, *P*_*cor*_ = 0.0842 in additive and dominant models, Table 3). T allele of rs662 and C allele of rs705379 were the risk alleles for higher carotid plaque score (rs662, TT + TC *vs* CC: 1.76 ± 1.60 *vs* 1.07 ± 0.97; rs705379, TT + TC *vs* CC: 1.22 ± 1.01 *vs* 1.79 ± 1.82). When the two SNPs were combined, the haplotype window rs662-rs705379 in *PON1* had a significant association with carotid plaque score (*P*_*cor*_ < 0.05, Table 4). TC haplotype of rs662-rs705379 posed a higher risk for higher carotid plaque score (unstandardized coefficients = 0.0873, *P*_*cor*_ < 0.05). None of other SNPs and haplotypes showed significant association with carotid plaque score (*P*_*cor*_ > 0.05, Tables 3 and 4).

**Table 3:** **Genotypes, minor allele frequency, Hardy-Weinberg Equilibrium and single-marker association analysis in all selected SNPs**

**Genes**	**SNPs**	**Alleles** ^**a**^ **(1/2)**	**Genotypes (11/12/22)**	**MAF**	**HWE**	**Corrected** ***P*** **values** ^**b**^			**1-β**	**SS**_**0.8**_
						**Additive**	**Dominant**	**Recessive**		
*SOD2*	rs4880	G/A	4/24/100	0.1250	0.1065	0.9588	0.9588	0.9588	0.0230	1371
*SOD3*	rs2536512	A/G	10/60/58	0.3125	0.4103	0.9588	0.9609	0.9588	0.0030	7785
*CAT*	rs769217	T/C	29/69/30	0.4961	0.4792	0.9588	0.9657	0.9588	0.0040	5862
*PON1*	rs662	T/C	15/49/64	0.3086	0.2986	0.0842	0.0528	0.9588	0.5240	194
	rs705379	T/C	35/49/44	0.4648	0.0124	0.0842	0.0842	0.4988	0.3020	283
*PPARG*	rs3856806	T/C	8/43/77	0.2305	0.6168	0.9588	0.9588	0.9588	0.0130	2072
*ADIPOQ*	rs1501299	T/G	8/56/64	0.2812	0.5107	0.9657	0.9588	0.9588	0.0030	8510
	rs2241766	G/T	9/59/60	0.3008	0.3994	0.9657	0.9657	0.9588	0.0050	5443
*IL10*	rs1800872	G/T	17/47/64	0.3164	0.1012	0.8279	0.9588	0.5357	0.0660	684
*TGFB1*	rs1800469	G/A	20/61/47	0.3945	1	0.9588	0.9588	0.9588	0.0100	2632
	rs1800470	A/G	21/61/46	0.4023	1	0.9588	0.9588	0.9588	0.0220	1404
	rs4803455	A/C	20/50/58	0.3516	0.1203	0.9588	0.9588	0.9588	0.0080	3085
*NOS3*	rs1799983	T/G	1/29/98	0.1211	0.6926	0.9657	0.9813	0.9588	0.0040	5862

^a^Allele 1 is the minor allele and allele 2 is the major allele.

^b^Corrected *P* values after false discovery rate correction for multiple testing (including the 39 tests in the single-marker association analyses between the 13 SNPs and carotid plaque score across the three genetic models).

Post-RT duration and number of cardiovascular risk factors were adjusted using linear regression in all association analyses. Statistical power (1-*β*) was calculated using the model with smallest observed *P* value. SS_0.8_ was the sample size to achieve a statistical power of 0.8 in the corresponding analysis. MAF, minor allele frequency; HWE, Hardy-Weinberg Equilibrium.

**Table 4:** **Association analyses between haplotypes and carotid plaque score in post-RT NPC patients**

**Genes**	**D’**	**r** ^**2**^	**Hap**	**Freq**	***P***_***cor***_	**1-β**	**SS**_**0.8**_
*PON1*							
rs662-rs705379	0.871	0.294	TC	0.290	0.0158	0.849	117
			CT	0.446	0.1881		
			TT	0.019	0.6840		
			CC	0.245	0.9740		
*ADIPOQ*							
rs1501299- rs2241766	1	0.168	GG	0.301	0.9740	0.047	1047
			TG	0.418	0.9740		
			TT	0.281	0.9740		
*TGFB1*							
rs4803455- rs1800470	0.88	0.628	CG	0.573	0.8766	0.033	1363
			AA	0.326	0.8766		
			AG	0.025	0.8766		
			CA	0.076	0.9740		
rs1800470-rs1800469	1	0.936	AG	0.391	0.8766	0.061	875
			GA	0.594	0.8766		
			AA	0.012	0.9740		
rs4803455-rs1800470-rs1800469	0.883	0.648	AAG	0.322	0.8766	0.097	638
			CGA	0.573	0.8766		
			CAG	0.069	0.9740		
			AGA	0.021	0.9740		

Five haplotype windows (HWs) include rs662-rs705379, rs1501299-rs2241766, rs4803455-rs1800470, rs1800470-rs1800469, and rs4803455-rs1800470-rs1800469. Post-RT duration and number of cardiovascular risk factors were adjusted using linear regression in all analyses. Multiple testing was corrected by false discovery rate. Statistical power (1-β) was calculated using the model with smallest observed *P* value. SS_0.8_ was the sample size to achieve a statistical power of 0.8 in the corresponding analysis. Hap, haplotype; Freq, frequency; *P*_*cor*_ *, Corrected P* value*.*

## Discussion

Carotid atherosclerosis is a common complication in post-RT NPC patients. However, the mechanisms of radiation-induced carotid atherosclerosis are still unclear and no previous study has reported the association between genetic polymorphisms and radiation-induced carotid atherosclerosis. The present study comprehensively investigated the association between 13 SNPs in anti-atherosclerotic genes and radiation-induced carotid atherosclerosis. Results showed that SNPs in *PON1* tended to be genetically associated with carotid plaque score in post-RT NPC patients.

PON1 is one of the important enzymes for hydrolyzing LDL oxidation, playing a pivotal role against carotid atherosclerosis. The SNP rs662 (T > C) locating in the coding region of *PON1* gene replaces glutamine (Q) by arginine (R) at codon 192 (Q192R). This variation affects the activities of PON1 in the hydrolysis of different substrates. The 192Q allozyme has higher hydrolytic activity toward diazoxon, soman and sarin, while the 192R allozyme is more efficient for hydrolyzing paraoxon and fenitroxon [35,36]. Another important variation, rs705379, is located at position −107 of the promoter region (−107 T/C), which contributes to a decrease in the *PON1* expression level and PON1 circulating concentration [37].

In the present study, significant observed *P* values were found in the association analyses between carotid plaque score and rs662 as well as rs705379 in additive and dominant models (rs662, *P* = 0.0054 and 0.0014 respectively; rs705379, *P* = 0.0077 and 0.0086 respectively). The significant association failed to survive in the correction for multiple testing by FDR, but it was close to be significant (*P*_*cor*_ = 0.0528 and 0.0842 respectively). In the association analyses with rs662 and rs705379, the statistical power was 0.524 and 0.302 respectively. To achieve a statistical power of 0.8, at least 194 and 283 patients would be needed respectively. Therefore, small sample size in the present study (n = 128) may account for the non-significant findings. Future studies with larger sample sizes are needed for investigating the association. Nevertheless, the two SNPs had a cumulative effect on carotid plaque score. Patients carrying T allele in rs662 (QR + QQ, n = 64) had 1.76 ± 1.60 of carotid plaque score, whilst those with CC genotype in rs705379 (n = 44) had 1.79 ± 1.82 of carotid plaque score. In patients carrying both T allele in rs662 and CC genotype in rs705379 (n = 38), the plaque score was 1.94 ± 1.89. TC haplotype in rs662-rs705379 showed significant association with the plaque score after the correction for multiple testing (*P*_*cor*_ = 0.0158). Thus, rs662 and rs70539 in combination were more powerful to detect the association with carotid plaque score in the present study. TC in rs662-rs705379 would be the risk haplotype for carotid plaque score in post-RT NPC patients.

D’ and r^2^ of the two SNPs were 0.871 and 0.294 respectively. The high D’ indicated that the two SNPs were in LD and were co-inherited most of the time. However, the different frequencies of alleles in the two SNPs (minor allele frequency = 0.3086 and 0.4648 respectively) resulted in the low r^2^. Therefore, the two SNPs cannot predict for each other.

In contrast to previous studies in which the R variant of the Q192R polymorphism (C allele in rs662) was a risk factor for spontaneous atherosclerosis diseases [21,38], the present study found that the R variant would be protective for radiation-induced carotid atherosclerosis. Patients carrying RR had lower plaque score as compared to those with QR and QQ genotypes (RR *vs* QR + QQ: 1.07 ± 0.97 *vs* 1.76 ± 1.60). In an in vitro model, Aviram *et al.* documented that RR and QQ allozymes serve anti-oxidant activities in different stages based on different substrates [39]. The various hydrolytic activities of R and Q allozymes in PON1 for different substrates may account for the conflicting results between the present and previous studies. The oxidative condition in the irradiated cases may be different from that in those without radiation treatment. PON1 may protect LDL from oxidation by a certain activity based on the different substrates in the irradiated cases and the non-irradiated ones. Thus, RR of Q192R polymorphism would be protective against radiation-induced carotid atherosclerosis, although it may be the risk of spontaneous atherosclerosis diseases.

### Limitations

The present study was a cross-sectional investigation with only 128 post-RT NPC patients and without a non-irradiated control group. The study design and small sample size limited the statistical power in association analyses. Thus, the potential association between some SNPs and carotid plaque score in post-RT NPC patients, and the potential interaction between the TC haplotype and irradiation, may not be fully investigated. In addition, the present study did not investigate the PON1 concentration and activity due to limited resources. Whether the TC haplotype decreases the concentration and activity of PON1 and consequently promotes the plaque score in post-RT NPC patients remains to be confirmed in future studies. Given that this study was an exploratory investigation, and the sample size and study design were limited, future investigations, especially prospective studies, are suggested to investigate the role of PON1 in the development of radiation-induced carotid atherosclerosis and the effectiveness of using the TC haplotype in the prevention of radiation-induced carotid atherosclerosis.

## Conclusion

TC haplotype in rs662-rs705379 of *PON1* is likely to be a genetic risk factor of carotid plaque score. The present study provides preliminary finding of the association between genetic polymorphisms with the radiation-induced carotid atherosclerosis in post-RT NPC patients, which facilitates the understanding of genetic markers for the selection of NPC patients with high risk of carotid atherosclerosis so as to conduct appropriate examinations for early diagnosis and prompt treatment for carotid atherosclerosis in post-RT NPC patients.
